# FFA-GPT: an automated pipeline for fundus fluorescein angiography interpretation and question-answer

**DOI:** 10.1038/s41746-024-01101-z

**Published:** 2024-05-03

**Authors:** Xiaolan Chen, Weiyi Zhang, Pusheng Xu, Ziwei Zhao, Yingfeng Zheng, Danli Shi, Mingguang He

**Affiliations:** 1 School of Optometry, The Hong Kong Polytechnic University, Kowloon, Hong Kong, China; 2 State Key Laboratory of Ophthalmology, Zhongshan Ophthalmic Center, Sun Yat-sen University, Guangdong Provincial Key Laboratory of Ophthalmology and Visual Science, Guangdong Provincial Clinical Research Center for Ocular Diseases, Guangzhou, China; 3 Research Centre for SHARP Vision (RCSV), The Hong Kong Polytechnic University, Kowloon, Hong Kong, China; 4 Centre for Eye and Vision Research (CEVR), 17W Hong Kong Science Park, Hong Kong, China

**Keywords:** Medical imaging, Retinal diseases

## Abstract

Fundus fluorescein angiography (FFA) is a crucial diagnostic tool for chorioretinal diseases, but its interpretation requires significant expertise and time. Prior studies have used Artificial Intelligence (AI)-based systems to assist FFA interpretation, but these systems lack user interaction and comprehensive evaluation by ophthalmologists. Here, we used large language models (LLMs) to develop an automated interpretation pipeline for both report generation and medical question-answering (QA) for FFA images. The pipeline comprises two parts: an image-text alignment module (Bootstrapping Language-Image Pre-training) for report generation and an LLM (Llama 2) for interactive QA. The model was developed using 654,343 FFA images with 9392 reports. It was evaluated both automatically, using language-based and classification-based metrics, and manually by three experienced ophthalmologists. The automatic evaluation of the generated reports demonstrated that the system can generate coherent and comprehensible free-text reports, achieving a BERTScore of 0.70 and F1 scores ranging from 0.64 to 0.82 for detecting top-5 retinal conditions. The manual evaluation revealed acceptable accuracy (68.3%, Kappa 0.746) and completeness (62.3%, Kappa 0.739) of the generated reports. The generated free-form answers were evaluated manually, with the majority meeting the ophthalmologists’ criteria (error-free: 70.7%, complete: 84.0%, harmless: 93.7%, satisfied: 65.3%, Kappa: 0.762–0.834). This study introduces an innovative framework that combines multi-modal transformers and LLMs, enhancing ophthalmic image interpretation, and facilitating interactive communications during medical consultation.

## Introduction

Fundus fluorescein angiography (FFA) is a valuable diagnostic imaging technique that uses the administration of a fluorescent dye to evaluate the retinal circulation and visualize blood flow. Compared to other imaging modalities such as fundus photography, FFA has significant advantages in evaluating various vascular ocular diseases, such as diabetic retinopathy (DR), central serous chorioretinopathy (CSC), and retinal vein occlusion (RVO), as it provides more detailed and dynamic vascular images^1^. However, the increased level of detail in the images also means a more challenging interpretation. The interpretation of FFA images requires extensive professional expertise and comprehensive training in ophthalmology, which may lead to a shortage of qualified FFA reports in regions lacking retinal specialists^2^. Additionally, ophthalmologists frequently dedicate a substantial amount of time and effort to writing medical reports in conventional clinical practice. There is an urgent need for intelligent tools to effectively manage these challenges.

Image-to-text conversion technology holds the potential to bridge this gap and has made significant strides in the realm of natural images^3^. Previous research has explored the application of these techniques in generating reports using FFA image data^4,5^. However, these studies have limitations in terms of comprehensive evaluation by ophthalmologists. Additionally, they have focused solely on report generation and overlooked the importance of providing further explanation after generating the FFA reports, a critical step in enhancing patient comprehension of clinical information and saving valuable time for doctors. Due to the specialized nature of these reports, patients often struggle to understand the content fully, leading to difficulties in addressing consultation questions and necessitating additional face-to-face consultations. In such cases, having a model that can handle general consultation questions would be beneficial, as it would save valuable time for doctors and enable more patients to receive professional medical services.

Recent astonishing advancements in large language models (LLM) have brought forth new possibilities for addressing these challenges^6^. Previous studies have evaluated the performance of different LLMs in ophthalmic question-answering (QA) tasks. For instance, Cai et al.^7^ assessed the performance of ChatGPT in ophthalmology using board–style questions and found that ChatGPT-3.5 provided correct answers in 58.8% of the questions, while ChatGPT-4.0 achieved a higher accuracy of 71.6%. Another study showed that GPT4-V(ision) model falls short when addressing QAs for ophthalmic images, with the best performance on Slit lamp images only reaching an accuracy of 42.0%^8^. Llama 2^9^, the latest development in open-source LLMs, is renowned for its flexibility and scalability. It holds promise in various medical applications^10–12^, including ophthalmology, where it can offer valuable medical advice and assistance for ophthalmic consultations^13^. However, the performance of responses generated by LLMs in addressing queries related to ophthalmology reports has not yet been evaluated. Currently, there is also no artificial intelligence (AI) system capable of effectively integrating FFA image-text information and providing QA interactions.

To maximize the AI-assisted interpretation of FFA images, our goal is to develop a two-stage FFA-GPT system. By combining the capabilities of image-text conversion models and LLMs, we aim to achieve FFA report generation and subsequent interactive QA, thereby reducing the reliance on retinal specialists.

## Results

**Fig. 1:**
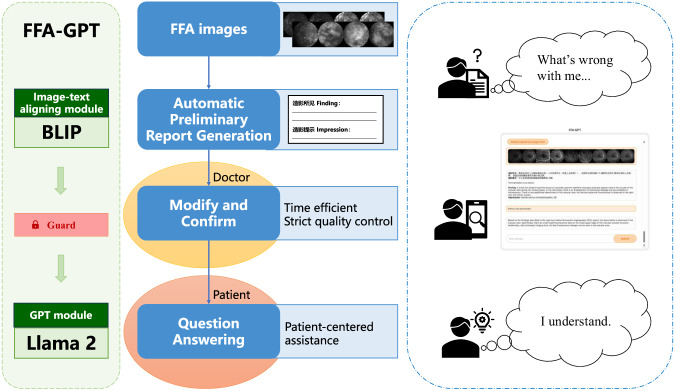
Schematic diagram of this study. FFA fundus fluorescein angiography, GPT generative pre-trained transformer, BLIP bootstrapping language-image pre-training.

The study flow chart is shown in Fig. 1. The final dataset used in the study consisted of 654,343 FFA images paired with 9392 reports. Among these images, 421,916 (64.5%, with 6312 reports) were for training, 76,900 (11.8%, with 1052 reports) were for validation, and 155,527 (23.8%, with 2028 reports) were for testing. The age of the participants had a median value of 51 years, with an interquartile range of 36 to 62 years. Of the participants, 5190 (55.3%) were male. More detailed characteristics of the dataset can be found in Table 1.

**Table 1:** Fundus fluorescein angiography dataset characteristics

	Total	Train	Validation	Test	*P* value
**Population**					
No.	9392	6312	1052	2028	
Age, median (IQR)	51 (36, 62)	51 (37, 62)	51 (36, 61)	50 (33, 62)	0.029
Sex, n (%)					0.115
Female	4202 (44.7)	2795 (44.3)	502 (47.7)	905 (44.6)	
Male	5190 (55.3)	3517 (55.7)	550 (52.3)	1123 (55.4)	
**FFA images**					
No.	654,343	421,916	76,900	155,527	
Phase^a^, n (%)					<0.001
Arterial	39,844 (6.1)	27,738 (6.6)	4794 (6.2)	7312 (4.7)	
Venous	372,725 (57.0)	239,211 (56.7)	43,778 (56.9)	89,736 (57.7)	
Late	241,774 (36.9)	154,967 (36.7)	28,328 (36.8)	58,479 (37.6)	

^a^Arterial: 30 to 60 seconds; Venous: 1 to 5 minutes; Late: 5 to 10 minutes.

IQR=Interquartile Range, FFA=fundus fluorescein angiography.

The eye conditions extracted from the original FFA reports encompassed a wide range of conditions commonly encountered in clinical practice. The top five most prevalent conditions were media opacity (11.5%), proliferative diabetic retinopathy (8.5%), macular edema (7.8%), DR (7.7%), and cystoid macular edema (7.4%). Additionally, the dataset included several rare diseases. A comprehensive overview of the main conditions can be found in Table 2.

**Table 2:** The main eye conditions extracted from the fundus fluorescein angiography reports (total *N* = 9392)

Conditions	*N* (%)
Media opacity	1078 (11.5%)
Proliferative diabetic retinopathy	802 (8.5%)
Macular edema	729 (7.8%)
Diabetic retinopathy	720 (7.7%)
Cystoid macular edema	699 (7.4%)
Congenital retinal fold	374 (4.0%)
Choroidal neovascularization	338 (3.6%)
Branch retinal vein occlusion	288 (3.1%)
Severe nonproliferative diabetic retinopathy	232 (2.5%)
Uveitis	225 (2.4%)
Unremarkable changes	225 (2.4%)
Central serous chorioretinopathy	206 (2.2%)
Diabetic maculopathy	182 (1.9%)
Diabetic macular edema	182 (1.9%)
Pathologic myopia	175 (1.9%)
Nonproliferative diabetic retinopathy	164 (1.7%)
Central retinal vein occlusion	157 (1.7%)
Wet age-related macular degeneration	153 (1.6%)
Polypoidal choroidal vasculopathy	149 (1.6%)
Retinal dystrophy	116 (1.2%)
Retinitis pigmentosa	111 (1.2%)
Vasculitis	110 (1.2%)
Epiretinal membrane	109 (1.2%)
Familial exudative vitreoretinopathy	106 (1.1%)
Vitritis	106 (1.1%)
Panuveitis	92 (1.0%)
Intermediate uveitis	67 (0.7%)
Myopia	59 (0.6%)
Age-related macular degeneration	54 (0.6%)
Choroidal mass	52 (0.6%)
Retinal telangiectasia	51 (0.5%)

### Automatic evaluation

The FFA-GPT model demonstrated satisfactory performance in generating FFA reports on the testing set, as evaluated using both language-based metrics and classification-based metrics (see Table 3). For language-based metrics, the model achieved the following scores: Bilingual Evaluation Understudy (BLEU)1 = 0.48, BLEU2 = 0.42, BLEU3 = 0.38, BLEU4 = 0.34, Consensus-based Image Description Evaluation (CIDEr)=0.33, Recall-Oriented Understudy for Gisting Evaluation (ROUGE) = 0.36, Semantic Propositional Image Caption Evaluation (SPICE) = 0.18, and Bidirectional Encoder Representations from Transformers Score (BERTScore)=0.70. These metrics assess the quality and similarity of the generated reports compared to the reference reports. For the classification-based metrics, we extracted conditions from the generated reports using a keyword-matching approach and categorized them individually. We then calculated the classification-based metrics for each condition based on the keyword-matching results and ranked all the conditions accordingly. The top-5 conditions with the highest F1 scores, which were microaneurysm, DR, arteriosclerosis, laser spots, and scar, were selected to demonstrate the model’s ability to classify multiple conditions. These conditions exhibited high specificities, all above 0.94. The accuracy ranged from 0.88 to 0.93. The F1 scores for these conditions were 0.82, 0.80, 0.73, 0.66, and 0.64, respectively, indicating a strong overall performance in disease classification. Based on previous studies^2,14–19^, we also explored the classification performance of nine commonly diagnosed conditions using FFA (see Supplementary Table 1). It can be observed that the model exhibited acceptable classification performance for DR and DR-related lesions, such as microaneurysm and laser scar.

**Table 3:** Model performance in the test set (155,527 images with 2028 reports)

A							
BLEU1	BLEU2	BLEU3	BLEU4	CIDEr	ROUGE	SPICE	BERTScore
0.48	0.42	0.38	0.34	0.33	0.36	0.18	0.70

B					
Conditions	Accuracy	Specificity	Precision	Sensitivity	F1 score
Microaneurysm	0.91	0.96	0.76	0.89	0.82
Diabetic retinopathy	0.93	0.97	0.74	0.88	0.80
Arteriosclerosis	0.88	0.94	0.69	0.77	0.73
Laser spots	0.91	0.95	0.63	0.69	0.66
Scar	0.90	0.95	0.63	0.66	0.64

A. Language-based metrics. B. Multi-class condition classification by participant.

Furthermore, we conducted an investigation to examine how changing the number of input images impacts the performance of the model. The number of input images ranged from 1 to 12. We observed that most of the metrics gradually improved as the number of input images increased, while BERTScore remains stable at around 0.70. However, the performance gains reached a plateau when at least two images were provided, particularly for the BLEU1, ROUGE, and SPICE metrics. The performance of the model showed greater volatility when measured by the CIDEr metric. Notably, the optimal value of 0.36 was achieved only when the number of input images exceeded 11 (see Supplementary Fig. 1).

### Manual evaluation

**Fig. 2:**
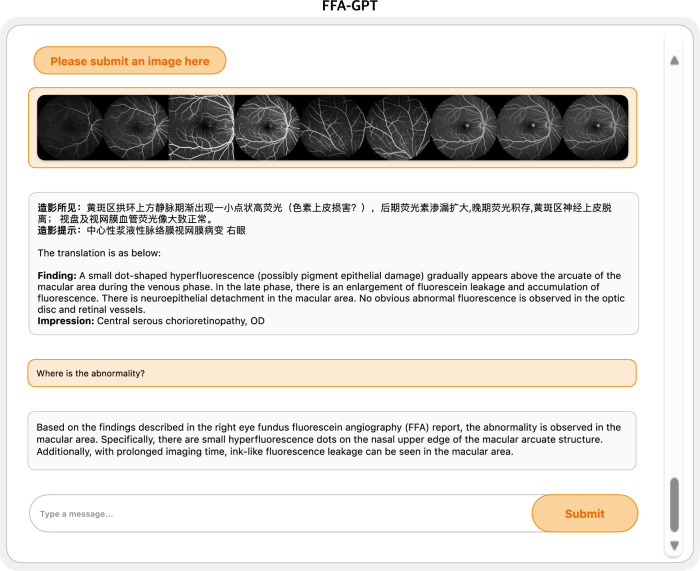
Demonstration of the integrated interface of our model. FFA fundus fluorescein angiography, GPT generative pre-trained transformer, OD right eye.

During the analysis of the clinical applicability of FFA-GPT by ophthalmologists, we observed promising quality in both the model-generated reports and answers. Figure 2 showcases an example of the integrated interface of our model.

**Fig. 3:**
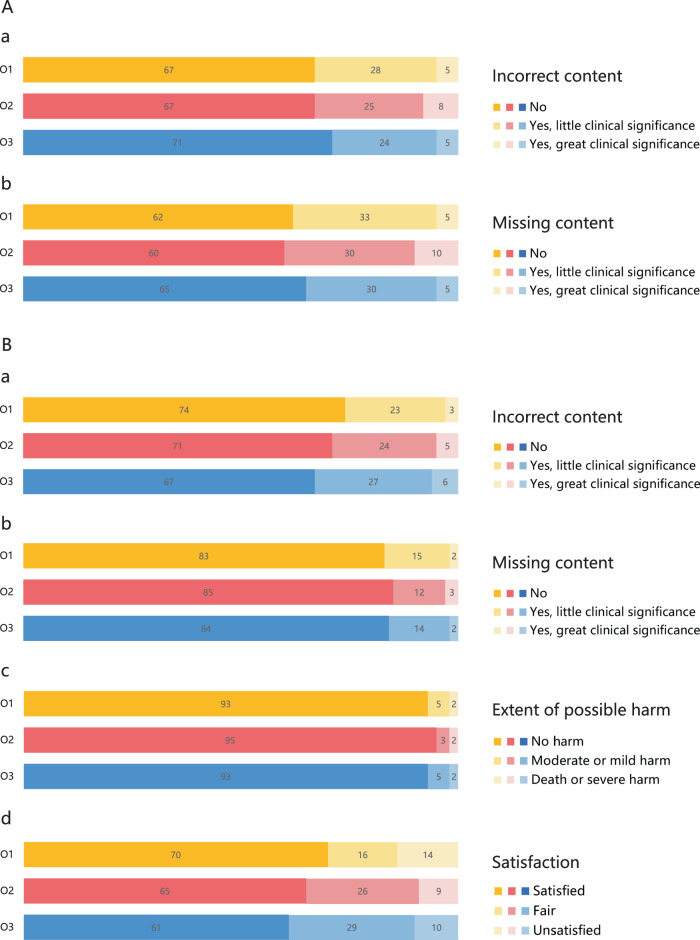
Human evaluation. **A** Report generation. **B** Question answering. O Ophthalmologist.

The quality of the reports generated by the FFA-GPT model is comparable to the ground truth reports. Fleiss’ kappa values indicate a good agreement between the three graders for assessing report quality, with a kappa value of 0.746 for completeness and 0.739 for accuracy, respectively (see Supplementary Table 2). Figure 3A summarizes the scoring of each rater on the generated reports in two dimensions. On average across all three raters, the generated reports with no incorrect content in 68.3% of cases, indicating the accuracy of the reports in describing the anatomical location of lesions and fluorescent imaging characteristics. Reports with minor errors accounted for 25.7%, while reports with significant errors accounted for 6.0%. These errors were mainly attributed to inaccuracies in describing fluorescence-like features and misidentification of complex lesions, such as choroidal mass. In terms of completeness, approximately 62.3% of the generated reports had no missing content. In 31.0% of the reports, there were minor instances of missing content, such as extremely small lesions. However, 6.7% of reports had a significant amount of missing content, primarily due to unclear imaging leading to false negatives. Examples of different grades of generated reports are shown in Supplementary Fig. 2.

After the first stage evaluation of report generation, we identified 20 out of 31 conditions (approximately 64.5%) that received positive feedback in the professional assessment. These cases meet the criteria to proceed through the Guard mechanism and can be forwarded into the Generative Pre-trained Transformer (GPT) module. The negative examples and their manually adjusted versions through the Guard mechanism, as well as subsequent QA examples based on these reports, are provided in Supplementary Fig. 3. It can be observed that the unadjusted report missed important lesions, and QA based on the error report could mislead patients. This demonstrates that automatic report generation simply serves as an assistance, the report distributed to patients cannot be fully automatic without the supervision of ophthalmologists. Out of the 20 question sets, we randomly selected 5 questions and combined them with the 20 approved cases to create 100 QA pairs, representing 20 distinct diseases. This dataset serves as the evaluation set for QA.

The evaluation results of the generated answers showed an average of 70.7% of the responses were virtually error-free, 24.7% had minor errors, and 4.6% had major errors for accuracy. In terms of completeness, an average of 84.0% of the answers provided complete responses to the questions related to the reports, 13.7% had minor omissions, and only 2.3% had significant omissions. Regarding the potential harm caused by the answers, the majority (over 93.7%) were deemed safe. However, two answers were identified as potentially causing severe harm. One answer suggested incorrect hormone therapy for CSC patients, while another suggested unreliable at-home light therapy for patients with RVO. In terms of satisfaction, the model achieved a satisfaction rate of 65.3%. The Fleiss’ kappa of the three raters ranged from 0.762 to 0.834, indicating substantial to almost perfect agreement among the raters, highlighting the reliability of the scoring process. The scoring details of each rater for the generated answers and the consistent results among them can be found in Fig. 3B and Supplementary Table 3, respectively. Examples of generated answers with great clinical significance in accuracy, completeness, harmfulness, and unsatisfied are provided in Supplementary Table 4.

## Discussion

In this study, we developed a dual-task model for ophthalmic image analysis. Firstly, we utilized a multimodal transformer to intelligently convert FFA images into medical reports. Then, we implemented an LLM to facilitate interactive QA. Through comprehensive automated and manual evaluations, the system demonstrated reliable and satisfactory performance. This research represents the demonstration of the large language model’s potential as an assistant for generating medical reports and enabling QA tasks to enhance FFA image interpretation.

Previous studies have developed AI models based on FFA image data. Some of these studies have focused on detecting multiple conditions in FFA images, such as DR^15,16^ and CSC^14^. Other studies have aimed to automatically generate FFA reports to address gaps in ophthalmic image-to-text conversion. Li et al.^5^ introduced a cross-modal clinical graph model for generating ophthalmic reports using 330,461 images. Lin et al.^4^ introduced contrastive pre-training to medical report generation based on datasets with multi-modalities, including FFA. Compared to these studies, FFA-GPT offers several advantages. Firstly, our model combines disease classification and report generation capabilities, accurately identifying retinal conditions, particularly DR, and generating coherent and comprehensible free-text reports. However, further improvements are required to enhance the model’s performance in classifying conditions other than DR. Secondly, to evaluate the expertise of our model, we collected clinically annotated reports as references and involved ophthalmic experts to assess the professional performance of the model in terms of accuracy and completeness. Over 60% of the reports met the clinical requirements, whereas Li et al.‘s study only achieved 44.7%. Additionally, our model can accept a random number of input images, which is more feasible in real-world applications. Lastly, in the actual clinical setting, patients often have many questions even after receiving the report, which require ophthalmologists’ explanations. FFA-GPT not only generates reports but also provides interactive explanations in the second stage, making it a comprehensive solution. Future research should focus on enhancing the quality and accuracy of the generated reports, thereby further minimizing the time for manual intervention. This can be achieved by incorporating more diverse data and investigating and comparing different architectural choices to identify the optimal framework^5,20^. Moreover, prospective clinical trials could be considered to validate the model’s effectiveness in improving report efficiency within real-world clinical settings.

Doctor-patient interaction is a crucial aspect of healthcare^21^. Patients often have limited knowledge of medical concepts and terminology, and it is the doctor’s responsibility to communicate complex medical information understandably while addressing the patient’s needs and concerns. Conversational large language models like ChatGPT and Llama 2 offer an alternative approach for effective AI-human interaction and collaboration. The integration of LLMs into the image-to-text conversion framework offers several advantages. Firstly, the LLM functions as an intelligent scheduler, activating the image reading module based on the context. This allows for the combination of text reports, which contain crucial details of patient FFA imaging results, with the world knowledge of the GPT module in LLM. This effectively mitigates LLM’s hallucinations and generates optimal answers. Secondly, following a comprehensive evaluation, we demonstrated that the LLM can effectively handle general inquiries related to ophthalmic image reports. The scarcity of high-quality medical QA data poses a real-world challenge. By incorporating the LLM into the report generation model, we introduced a solution for ophthalmic QA without the need for additional QA training. This approach avoids the issue of insufficient datasets and optimizes the utilization of computational resources. Thirdly, our two-stage framework offers greater interpretability than an end-to-end one. The separate generation process allows for increased transparency, and the embedded guard mechanism can assess the quality of the generated reports before their release. This ensures that only reports that have been rigorously reviewed and refined by medical professionals are made available for patients’ use in QA. This assures our system to interact with end-users while maintaining its social responsibility. Continued efforts in empowering LLMs with ophthalmic knowledge, such as incorporating retrieval-augmented generation techniques to include more resources for addressing ophthalmic tasks, will contribute to enhancing the model’s applicability. Additionally, improving the interpretability of the model would be beneficial.

Previous studies have demonstrated that AI assistance can be beneficial in reducing diagnostic time and improving radiologists’ performance^22,23^. In daily clinical care, our proposed FFA-GPT offers an automated pipeline for interpreting FFA images, both in the pre- and post-explanation stages. In the stage of generating specialized reports from FFA images, our model shows potential in generating preliminary reports to assist ophthalmologists in interpreting ophthalmic images. It has the potential to reduce diagnostic errors caused by difficulties and fatigue, thereby improving medical efficiency for ophthalmologists. Furthermore, in the stage of communicating with patients after generating specialized reports, our system is expected to assist patients in explaining medical concepts and procedures of FFA reports in simplified terms, potentially making it easier to understand. It can be embedded in applications and accessed through a mobile interface in the future. This enables us to provide additional information on common eye health issues and offer decision support during the consultation. However, the application of this model in a clinical environment requires careful consideration of ethical issues. Firstly, although our model safeguards patient data privacy through techniques such as de-identification and local model deployment, it is imperative to ensure that patients are fully informed and provide consent for the utilization of their data in AI model analysis during future prospective validation. Secondly, to enhance the interpretability of the model, future research development may consider incorporating techniques like Concept Relevance Propagation^24^ or visualization methods, thereby facilitating clinicians’ understanding of how the model analyzes images and makes assessments. It is also important to emphasize that such models are currently in the early stages and are intended to serve as auxiliary tools to assist ophthalmologists in improving their work efficiency and accuracy, rather than replace their judgment and decision-making. Therefore, the guard mechanism is crucial to promptly detect and correct model errors, ensuring clinical supervision and review of model outputs and minimizing the risk of misdiagnosis, thus ensuring feasibility and safety.

This research has several limitations. Firstly, there are potential risks associated with the generated medical models. Although our research has undergone comprehensive evaluations from multiple perspectives, validating the accuracy and correctness of the generated content still poses challenges. The phenomenon of hallucination stemming from LLM still exists in our model. Therefore, arming the LLM with ophthalmic knowledge and improved guard mechanisms may be important for future work in this regard. Secondly, multi-modal medical imaging has become one of the important trends in the field of medicine today, providing broader space and opportunities for the development of AI. Our model is currently limited to FFA images, and future research should focus on developing multi-modal imaging models. Lastly, our model lacks a fully external dataset for validation and instead relies on temporal-split data. Further evaluation and optimization of the model’s generalizability are needed for different spatial data and entirely new data.

In conclusion, this study introduces an innovative approach that combines a multi-modal transformer and an LLM to establish a connection between ophthalmic imaging and automated report generation, as well as facilitate interactive QA. The FFA-GPT system shows promising potential to enhance the interpretation and reporting of ophthalmic images, offering an important reference for the development of other image-based AI systems.

## Methods

### Dataset

Data for this study were retrospectively collected from a tertiary hospital in China from November 2016 to December 2019. To ensure patient privacy and adhere to ethical principles outlined in the Declaration of Helsinki, all patient data were anonymized. The study was approved by the Institutional Review Board of the Hong Kong Polytechnic University, and individual consent for retrospective analysis was waived. The dataset encompassed a broad spectrum of eye conditions, including DR, RVO, and CSC. The FFA images were captured using Zeiss FF450 Plus and Heidelberg Spectralis cameras from Heidelberg, Germany, with a resolution of 768 × 768 pixels. We excluded low-quality FFA images by extracting vessels^25^, where images with detectable vessel area ratios less than 0.005 were excluded. To facilitate external validation, the dataset was divided into training, validation, and testing subsets based on temporal splits. Specifically, images captured before June 2019 were allocated for training, while the remaining images captured after June 2019 were used for validation and testing. This temporal split strategy simulates a study where a model is developed using past data and subsequently validated and tested on future cases. It serves as a form of external validation, contributing to the robustness of the findings^26^.

### Development of FFA-GPT

We utilized the Bootstrapping Language-Image Pre-training (BLIP) framework as the image-text aligning module. BLIP is a pretrained architecture that has been trained on a vast dataset of natural images, which facilitated the acquisition of effective feature representations for both images and text^27^. Notably, the BLIP model demonstrates exceptional proficiency in encoding long-range dependencies, making it well-suited for handling intricate ophthalmic images and longer sequences of ophthalmic reports. Furthermore, the BLIP model possesses the capability to filter out noisy data during the training process, which substantially enhances the quality of large-scale training datasets. The framework comprises two key components: a visual transformer^28^ serving as the image encoder, and Bidirectional Encoder Representations from Transformers (BERT)^29^ serving as the language encoder and decoder.

To fine-tune the pre-trained BLIP model, we employed a dataset consisting of FFA images and their corresponding Chinese reports. During the training process, we randomly selected 1-9 images from each case as input for the model, ensuring a balanced representation of the arterial, venous, and late phases of FFA. All images were resized to 320×320 pixels. For the fine-tuning process, the model was trained with two NVIDIA Tesla V100 GPUs. The initial learning rate was set to 0.00002, with a weight decay of 0.05. We utilized AdamW^30^ optimizer with a cosine learning rate schedule. The fine-tuning was performed for 50 epochs and the whole training time was 3 days. The model with the highest BLEU1 score on the validation set was selected for testing.

It’s important to note that currently, automatic report generation is intended to assist doctors, rather than to produce reports that can be directly released to patients. Therefore, we included a guard mechanism for quality control of generated reports to reduce the risk of disseminating harmful information. This mechanism necessitates human evaluation and confirmation. Only reports that pass this confirmation or have been corrected by doctors can proceed to the next step. This guard mechanism is open to be optimized and updated to enhance efficiency.

Although the BLIP model can be used for further QA tasks, it requires a large amount of reliable QA training data and significant computational resources, which makes the training and continuous optimization challenging (see Supplementary Table 5). To enable QA without the need for fine-tuning and to facilitate interactive interpretation of the generated reports, we incorporated the advanced open-source large language model, Llama 2^9^ in the second step as the GPT module to enable QA. Specifically, this was achieved by giving instructions of ‘Please answer the following question based on the given FFA report’. We formulated a series of typical questions related to FFA reports, drawing on our clinical experience and following methods similar to those used by Momenaei et al.^31^. The question lists we created contain a total of 20 items covering a range of topics, including a summary of the reports, disease definitions, etiology, visual impact, prevention measures, further examinations, treatment options, prognosis, complications, and FFA testing information such as timing, specific phases, and post-test instructions (See Supplementary Table 6). Given that Llama 2 primarily excels in handling English data, we incorporated a translation plugin to create a connection between the two modules. These questions, along with the English translation of the generated reports, were then fed into the Llama 2 model for testing.

### Automatic evaluation of FFA-GPT

We conducted the automatic evaluation of the generated reports using language-based metrics and classification-based metrics^32^. The language-based metrics used in this study include BLEU, CIDEr, ROUGE, SPICE, and BERTScore. BLEU^33^ is a widely used automatic metric for evaluating the quality of machine translations and scores by calculating the overlap of n-grams between the generated text and reference texts. An n-gram is a contiguous sequence of ‘n’ words from a given sample of text. Given that many ophthalmic terms are compound words often composed of up to four words, we calculated BLEU1, BLEU2, BLEU3, and BLEU4 as evaluation metrics. CIDEr^34^ is developed for image captioning evaluation. It evaluates not just the presence of relevant words but also how frequently those words are used in similar contexts, providing higher scores for words that are both pertinent and rare. In our context, it helps to ensure that the terminology used is not only appropriate but also indicative of the distinctive features in the images, which is crucial for medical reporting. ROUGE^35^ focuses on the longest common subsequence between the generated text and reference texts, suitable for evaluating model performance at the sentence or paragraph level. Ophthalmic reports often require coherent descriptions of complex clinical information, and ROUGE helps to understand how well the model maintains coherence throughout the information presented. SPICE^36^ differs from BLEU by focusing on semantic accuracy in image caption generation rather than mere word or phrase matching, providing a comprehensive assessment of generated description quality. BERTScore^37^ utilizes the pre-trained BERT model to compute the semantic similarity between generated and reference text. It excels at assessing semantic match within the overall context, providing a nuanced and semantically oriented evaluation of model performance.

The classification metrics were employed to supplement the language-based metrics, providing a more comprehensive evaluation of the model’s ability to accurately identify diseases. Initially, we manually constructed a keyword dictionary for ophthalmic conditions. This dictionary included standard medical terms, root words, prefixes, and synonyms associated with each condition (sample shown in Supplementary Fig. 4). We then utilized this dictionary to automatically identify, extract, and standardize disease condition terminology present in the FFA reports. During the keyword-matching process, we also considered the presence of negations. We utilized regular expressions to check if the word was prefixed or followed by negation words such as ‘not observed’, ‘no apparent’, ‘without’, and ‘not supported’, and refrained from extracting the related disease terms. We employed a precise matching strategy while considering negation terms and the mapping is case-insensitive. These standardized terms were also used to evaluate the accuracy of disease identification in the generated reports. Based on the results of this keyword-matching process, we calculated classification metrics, including accuracy, sensitivity, specificity, precision, and F1 score. Accuracy is the most commonly used metric in classification tasks, representing the proportion of correctly predicted classifications out of the total predictions^38^. Sensitivity measures the proportion of actual positives correctly identified by the model. High sensitivity is crucial in medical diagnostics to ensure timely management of diseases by minimizing false negatives. Specificity is the proportion of actual negatives correctly identified and high specificity helps to ensure that only patients who truly have the disease receive further diagnosis and treatment. Precision indicates the proportion of positive identifications made by the model that are actually correct. F1 score is the harmonic mean of sensitivity and precision and provides a more comprehensive assessment of model performance, especially in situations of class imbalance^14,15,18,19^.

### Manual evaluation of FFA-GPT

To ensure the integrity and precision of our assessment, we conduct a human evaluation of reports generated by our model. For this evaluation, we randomly selected a subset of 100 images from the test set, and their corresponding generated reports were assessed by three experienced ophthalmologists (X.C., P.X., and Z.Z.) with an average of over five years of clinical experience. We followed a similar evaluation approach as Singhal K et al.^39^, focusing on two aspects: incorrect content and missing content. The aspect of incorrect content evaluates the model’s accuracy in report generation by determining whether the generated text contained any content that it should not. On the other hand, the aspect of missing content evaluates the model’s completeness in report generation by determining whether the generated text omitted any information that it should include.

The ophthalmologists independently rated the quality of the generated reports compared to the ground-truth reports for accuracy and completeness using a 3-level quality evaluation scale: ‘No,’ ‘Yes, little clinical significance,’ and ‘Yes, great clinical significance.’ To reduce the variability among the raters and ensure reliable results, we calculated Fleiss’ kappa^40^. Fleiss’ kappa is a measure of interrater agreement used to assess the consistency among multiple raters in a categorical classification task. It takes into account both the observed agreement and the agreement expected by chance, providing a statistical measure of agreement beyond random chance. The interpretation of its values varies as follows: 0-0.2 indicates slight agreement, 0.2-0.4 indicates fair agreement, 0.4-0.6 indicates moderate agreement, 0.6-0.8 indicates substantial agreement, and 0.8-1.0 indicates almost perfect agreement.

The answers to the report-related questions were also assessed by the three ophthalmologists using the same method mentioned in the manual evaluation of report generation. As there is no ground truth for the QA pairs, the evaluation criteria in this part are based on current scientific consensus and the ophthalmologists’ clinical experience. In addition to accuracy and completeness, we added two dimensions of harmfulness and satisfaction to the evaluation criteria^39^. Specifically, accuracy considered the correct identification of medical terms in the answers and the consistency with relevant medical recommendations and consensus. Completeness took into account not only direct answers to the inquiries but also necessary additional details relevant to clinical practice. The evaluation of harmfulness is due to the clinical recommendations that may prompt patients to take actual actions involved in the QA process, and the lack of human supervision. The raters considered the potential physical or mental health-related harm that may result from the actions prompted by the generated answer. The severity of harm was referenced by the Agency for Healthcare Research and Quality (AHRQ) common formats, and three options were provided: ‘No harm,’ ‘Moderate or mild harm,’ and ‘Death or severe harm.’ based on the anticipated impact on vision and physical health^41^. The severity of harm caused by answers is distinguished based on its direct or indirect impact on the patient’s future vision and physical health. It is important to note that while our rating was based on the AHRQ scale, it should be considered as a subjective assessment. The evaluation of satisfaction is crucial because relying solely on technical metrics, such as accuracy, may not fully capture users’ genuine needs and expectations. Also, the satisfaction assessment offered three options: Satisfied, Fair, and Unsatisfied. The inter-rater agreement was also calculated using Fleiss’ kappa, which is the same metric used in the evaluation of the generated reports.

### Supplementary information

Supplementary Figure 1-4 and Table 1-6

## Data Availability

Code is available at https://github.com/salesforce/BLIP and https://github.com/meta-llama/llama.
