# Supplementary material for: FFA-GPT: an automated pipeline for fundus fluorescein angiography interpretation and question-answer
Source: NPJ Digit Med. 2024 May 3;7:111. doi: 10.1038/s41746-024-01101-z (PMC11068733; doi:10.1038/s41746-024-01101-z)
Supplement: Supplementary file 1 — Supplementary Figure 1-4 and Table 1-6 [file 41746_2024_1101_MOESM1_ESM.pdf]

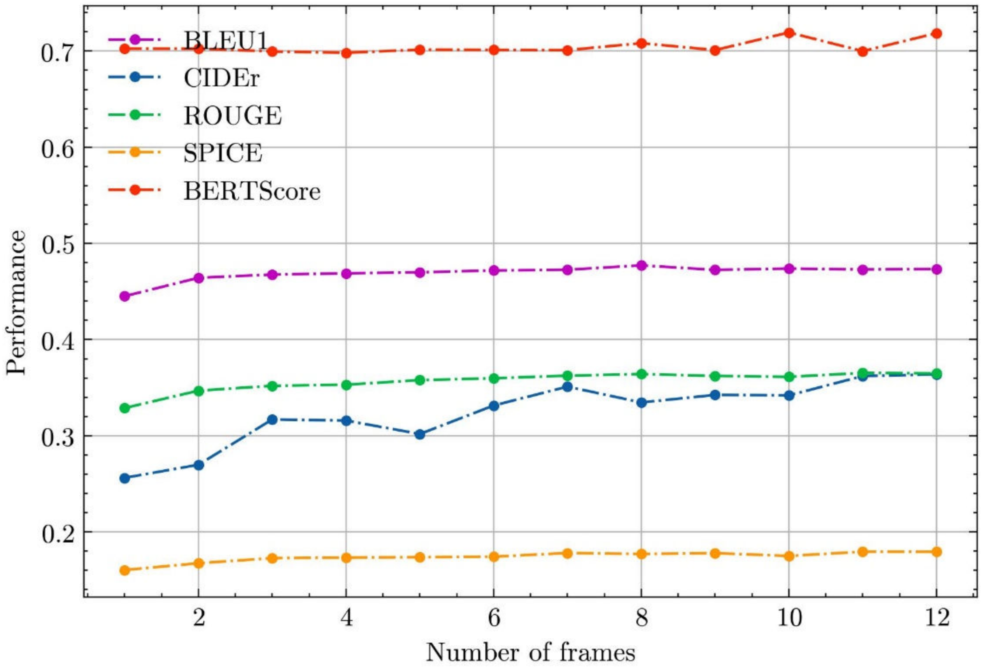


**Supplementary Figure 1. A. Impact of input FFA frame number on the report generation performance of FFA-GPT.** FFA=fundus fluorescein angiography, GPT=generative pre-trained transformer, BLEU=Bilingual Evaluation Understudy, CIDEr=Consensus-based Image Description Evaluation, ROUGE=Recall-Oriented Understudy for Gisting Evaluation, SPICE=Semantic Propositional Image Caption Evaluation, BERT=Bidirectional Encoder Representations from Transformers.

A

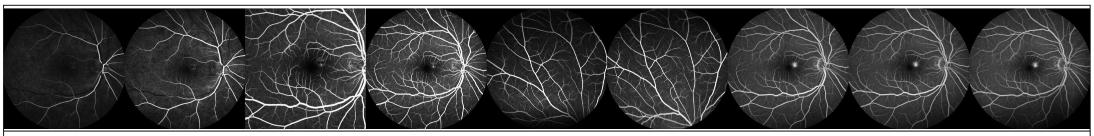

## Ground truth

**造影所见：**视网膜动静脉充盈回流时间大致正常；视盘未见明显异常荧光；黄斑区拱环结构完整，拱环鼻上缘可见点状高荧光，随造影时间延长，可见墨迹样荧光渗漏，各象限中周部未见明显异常荧光。

**造影提示：**中心性浆液性脉络膜视网膜病变 右眼

**Finding:** The retinal arteriovenous filling time is approximately normal. No apparent abnormal fluorescence is observed in the optic disc. The arcuate structure in the macular area is intact, with small hyperfluorescence dots on the nasal upper edge of the arcuate. With prolonged imaging time, ink-like fluorescence leakage can be seen. No obvious abnormal fluorescence is observed in the peripheral areas of each quadrant.

**Impression:** Central serous chorioretinopathy, OD

## Prediction

**造影所见：**黄斑区拱环上方静脉期渐出现一小点状高荧光（色素上皮损害？），后期荧光素渗漏扩大，晚期荧光积存，黄斑区神经上皮脱离；视盘及视网膜血管荧光像大致正常。

**造影提示：**中心性浆液性脉络膜视网膜病变 右眼

**Finding:** A small dot-shaped hyperfluorescence (possibly pigment epithelial damage) gradually appears above the arcuate of the macular area during the venous phase. In the late phase, there is an enlargement of fluorescein leakage and accumulation of fluorescence. There is neuroepithelial detachment in the macular area. No obvious abnormal fluorescence is observed in the optic disc and retinal vessels.

**Impression**: Central serous chorioretinopathy, OD

B

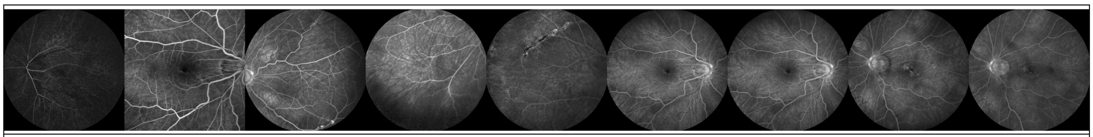

## Ground truth

**造影所见：**视盘晚期高荧光染色，边界清晰，视盘颞侧脉络膜萎缩弧；视网膜周边部血管通透性增加；黄斑区点状透见荧光；颞上、下方中周部可见带状色素上皮萎缩变性区透见荧光，间杂色素增殖遮蔽荧光。

**造影提示：**高度近视眼底改变

**Finding:** Optic disc shows clear boundaries with hyperfluorescence staining in the late stage. Peripapillary atrophy is observed in the temporal side of the optic. Increased vascular permeability is observed in the peripheral retinal vessels. Punctate transmitted fluorescence is seen in the macular area. In the temporal superior and inferior mid-periphery, there are areas of atrophic changes in the retinal pigment epithelium, showing transmitted fluorescence. The presence of scattered pigment proliferation shows blocked fluorescence.

**Impression:** High myopia.

## Prediction

**造影所见：**视盘荧光染色，视盘侧脉络膜萎缩；黄斑区早期即见环形弱荧光及低荧光，后期局部病灶荧光素渗漏扩大，晚期荧光积存；周边部部分血管通透性增加。

**造影提示：**高度近视眼底改变？黄斑区脉络膜新生血管？

**Finding:** Optic disc shows staining and choroidal atrophy. In the early stage, a ring-like weak fluorescence and low fluorescence are seen in the macular area. In the later stage, localized lesions show increased leakage of fluorescein and accumulation of fluorescence. Increased vascular permeability is observed in some peripheral vessels.

**Impression:** High myopia? Choroidal neovascularization in the macular area?

C

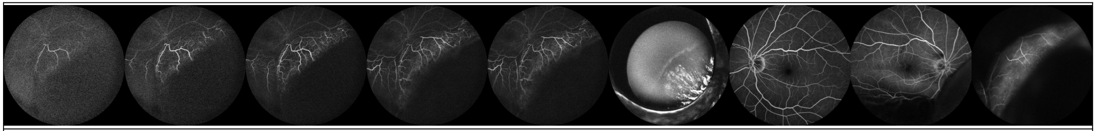

## Ground truth

**造影所见：**鼻下方脉络膜病变明显隆起；病变表面多灶性视网膜色素上皮损害，针尖状荧光素渗漏,病变强荧光染色；病变表面视网膜血管扩张、通透性增加；鼻下方周边部可见局限性睫状体脱离隆起；鼻侧及下方弥漫性视网膜神经上皮脱离，局部视网膜血管节段性通透性增加；视盘未见明显异常荧光；黄斑灶性视网膜色素上皮色素脱失，黄斑多灶性弱荧光渗出斑点。
**造影提示：**脉络膜占位性病变

**Finding:** Prominent elevation of choroidal lesions is observed below the nasal region. The surface of the lesions shows multifocal damage to the retinal pigment epithelium, pinpoint leakage of fluorescein, and the lesions show hyperfluorescence of staining. Dilated retinal vessels and increased permeability are observed on the lesion surface. Limited elevation of the ciliary body detachment is seen in the peripheral region below the nasal area. Diffuse detachment of the retinal pigment epithelium is observed on the nasal and below side, with segmental increases in vascular permeability in certain areas of the retina. No apparent abnormal fluorescence is observed in the optic disc. Focal depigmentation and multiple weak fluorescein leakage spots are seen in the macular area.
**Impression:** Choroidal space-occupying lesion.

## Prediction

**造影所见：**视盘及视网膜血管未见异常荧光；黄斑区下方点状色素上皮色素脱失透见荧光；中周部视网膜未见明显异常荧光。
**造影提示：**无。

**Finding:** No abnormal fluorescence is observed in the optic disc and retinal vessels. Punctate depigmentation is observed below the macular area, showing transmitted fluorescence. No apparent abnormal fluorescence is observed in the mid-periphery of the retina.
**Impression:** None.

**Supplementary Figure 2. Examples of generated reports in different grades.** A. No incorrect, missing content. B. With little clinical significance. C. With great clinical significance. The underlined text in the figures represents content that contains inappropriate information.

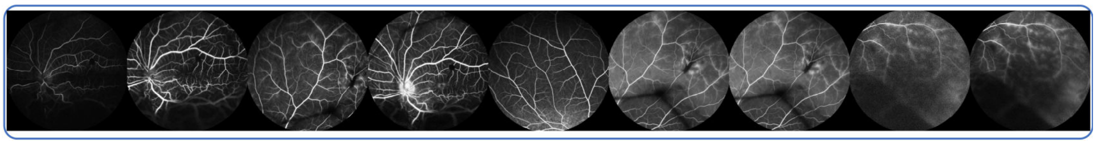

**Generated report & QA**

**Report:**

**造影所见：**视盘及视网膜血管未见异常荧光； 视网膜及黄斑区未见明显异常荧光。**造影提示：**无。

**Finding:** No abnormal fluorescence is observed in the optic disc and retinal vessels. No apparent abnormal fluorescence is observed in the retina and macula.

**Impression:** None.

**QA:**

**Q：** Is there any abnormality?

**A：** Based on the information provided in the finding, there is no abnormality observed in the optic disc, retinal vessels, retina, or macula. Therefore, the answer is no, there is no abnormality.

**Adjusted report & QA**

**Report:**

**造影所见：**视盘轻微渗漏，黄斑区见局部圆斑状遮蔽荧光；中周至周边视网膜脱离，伴荧光素渗漏。**造影提示：**左眼视网膜脱离

**Finding:** Mild leakage in the optic disc, with localized circular-shaped blocked fluorescence in the macular area. Retinal detachment is observed from the mid-periphery to peripheral retina, accompanied by leakage of fluorescein dye. **Impression:** Retinal detachment in the left eye.

**QA:**

**Q：** Is there any abnormality?

**A：** Based on the finding of mild leakage in the optic disc and localized circular-shaped blocked fluorescence in the macular area, as well as the observation of retinal detachment from the mid-periphery to peripheral retina, it is likely that there is an abnormality in the retina. The blocked fluorescence in the macular area may indicate a localized area of retinal degeneration or damage. The retinal detachment observed in the left eye may be due to a variety of causes, including retinal vein occlusion, retinal tears or retinal detachment. Further evaluation and testing may be necessary to determine the underlying cause and appropriate treatment.

**Supplementary Figure 3. The negative examples of reports not passing the Guard mechanism and their manually adjusted versions, along with examples of subsequent question answering based on both versions.** QA=question answering. Q=question. A=answer.

**Keyword dictionary**

**Disease 1:** Diabetic retinopathy.
**Abbr:** DR.
**Implication:** diabetic retinopathy.
**Keyword:** diabetic reti.
**Exclusion:** -.
**Finding:** MA.

**Disease 1.1:** Proliferative diabetic retinopathy.
**Abbr:** PDR.
**Implication:** proliferative diabetic retinopathy
**Keyword:** proliferative diabetic|DR4.
**Exclusion:** -.
**Finding:** retinal hemorrhage, RNV, NPA.

**Disease 1.2:** Nonproliferative diabetic retinopathy.
**Abbr:** NPDR.
**Implication:** nonproliferative diabetic retinopathy
**Keyword:** nonproliferative|background diabe|NPDR|DR2|DR3|DR1|non.*proliferative |BDR
**Exclusion:** -.
**Finding:** MA.

**Supplementary Figure 4. Samples of the keyword dictionary.** DR=diabetic retinopathy. MA=microaneurysm. PDR=proliferative diabetic retinopathy. RNV=retinal neovascularization. NPA=nonperfusion area. NPDR=nonproliferative diabetic retinopathy. BDR=background diabetic retinopathy.

**Supplementary Table 1. The performance of FFA-GPT on nine commonly diagnosed conditions using FFA.**

| **Conditions** | **Accuracy** | **Specificity** | **Precision** | **Sensitivity** | **F1 score** |
|---|---|---|---|---|---|
| Diabetic retinopathy | 0.93 | 0.97 | 0.74 | 0.88 | 0.80 |
| Microaneurysm | 0.91 | 0.96 | 0.76 | 0.89 | 0.82 |
| Laser scar | 0.91 | 0.95 | 0.63 | 0.69 | 0.66 |
| Hemorrhage | 0.78 | 0.90 | 0.38 | 0.55 | 0.45 |
| Retinal vein occlusion | 0.96 | 0.98 | 0.51 | 0.54 | 0.52 |
| Nonperfusion area | 0.90 | 0.99 | 0.10 | 0.53 | 0.16 |
| Central serous chorioretinopathy | 0.98 | 0.99 | 0.38 | 0.45 | 0.41 |
| Choroidal neovascularization | 0.96 | 0.98 | 0.27 | 0.34 | 0.30 |
| Uveitis | 0.95 | 0.99 | 0.23 | 0.56 | 0.33 |

**Supplementary Table 2. Qualitative model performance in the test set for report generation.**

A

| Incorrect Content | O1 | O2 | O3 | Kappa |
|---|---|---|---|---|
| **No** | 67% | 67% | 71% | |
| **Yes, little clinical significance** | 28% | 25% | 24% | 0.746 |
| **Yes, great clinical significance** | 5% | 8% | 5% | |

B

| Missing Content | O1 | O2 | O3 | Kappa |
|---|---|---|---|---|
| **No** | 62% | 60% | 65% | |
| **Yes, little clinical significance** | 33% | 30% | 30% | 0.739 |
| **Yes, great clinical significance** | 5% | 10% | 5% | |

**Supplementary Table 3. Qualitative model performance in the test set for medical question answering.**

A

| Incorrect Content | O1 | O2 | O3 | Kappa |
|---|---|---|---|---|
| **No** | 74% | 71% | 67% | |
| **Yes, little clinical significance** | 23% | 24% | 27% | 0.764 |
| **Yes, great clinical significance** | 3% | 5% | 6% | |

B

| Missing Content | O1 | O2 | O3 | Kappa |
|---|---|---|---|---|
| **No** | 83% | 85% | 84% | |
| **Yes, little clinical significance** | 15% | 12% | 14% | 0.806 |
| **Yes, great clinical significance** | 2% | 3% | 2% | |

C

| Extent of Possible Harm | O1 | O2 | O3 | Kappa |
|---|---|---|---|---|
| **No harm** | 93% | 95% | 93% | |
| **Moderate or mild harm** | 5% | 3% | 5% | 0.834 |
| **Death or severe harm** | 2% | 2% | 2% | |

D

| Satisfaction | O1 | O2 | O3 | Kappa |
|---|---|---|---|---|
| **Satisfied** | 70% | 65% | 61% | |
| **Fair** | 16% | 26% | 29% | 0.762 |
| **Unsatisfied** | 14% | 9% | 10% | |

**Supplementary Table 4. Examples of generated answers with great clinical significance in accuracy, completeness, harmfulness, and unsatisfied.** The underlined text in the table represents content that contains inappropriate information.

| Aspect | Report | Question | Answer |
|---|---|---|---|
| Accuracy | **Finding:** Left eye fundus fluorescein angiography (FFA): Arterial filling and venous return are mostly normal. There is increased vascularization of the retina, with vascular stiffness. Mild leakage is observed from clustered small vessels in the temporal side of the macula. Temporal, superotemporal, and inferotemporal regions show straightened and prematurely terminating blood vessels, with residual arcuate areas of non-perfusion. Within these non-perfusion areas, there are visible signs of old laser spots. On the nasal side, there are straightened blood vessels and circular laser spots. The optic disc shows patchy areas of hyperfluorescence in an arcuate spot.<br>**Impression:** Familial exudative vitreoretinopathy in the left eye. | What is the prognosis for this condition? | Based on the findings in the left eye, the prognosis for Familial Exudative Vitreoretinopathy (FEVR) is guarded. The increased vascularization of the retina, with vascular stiffness, suggests that the disease is progressing. <u>The mild leakage from clustered small vessels in the temporal side of the macula may indicate that the retinal layers are thinning</u>, which can lead to further vision loss. The straightened and prematurely terminating blood vessels in the temporal, superotemporal, and inferotemporal regions are a hallmark of FEVR and can lead to non-perfusion areas, which can result in permanent vision loss. <u>The presence of residual arcuate areas of non-perfusion and visible signs of old laser spots within these areas suggest that the disease has been present for some time.</u> The patchy areas of hyperfluorescence in the optic disc may indicate that the retina is still functioning, but the degree of damage is significant. <u>The presence of circular laser spots on the nasal side may indicate that previous laser treatment has been unsuccessful in stopping the progression of the disease.</u><br>Overall, the prognosis for FEVR is poor, with significant vision loss and potential for further progression of the disease. Early detection and treatment are crucial to minimize vision loss and improve the chances of preserving some vision. |
| Complete ness | **Finding:** There was an arteriovenous crossing sign beneath the optic disc, resulting in the distal branch vein showing delayed reflux in its side branch. Within the | What might be the cause of this condition? | Based on the findings described in the vascular ultrasound report, the likely cause of the branch retinal vein occlusion in the left eye is:<br>1. Arteriovenous crossing sign beneath the optic disc: This sign indicates the presence of a connection between the artery and vein beneath the optic disc, which can lead to venous congestion and retinal vein occlusion. |

| | | | |
|---|---|---|---|
| | affected area, there was venous congestion and leakage from the microvasculature, leading to the formation of numerous small non-perfused areas. Additionally, there was cystoid macular edema present along the arcades and its adjacent and below margins in the left eye. No significant abnormal fluorescence was observed in the optic disc of the left eye. **Impression**: Branch retinal vein occlusion Left eye. | | 2. Delayed reflux in the distal branch vein: The delayed reflux in the distal branch vein can cause venous congestion and lead to retinal vein occlusion.<br>3. Venous congestion and leakage from the microvasculature: The venous congestion and leakage from the microvasculature can lead to the formation of numerous small non-perfused areas in the affected eye.<br>4. Cystoid macular edema: The presence of cystoid macular edema in the left eye can cause vision loss and distortion.<br>5. No significant abnormal fluorescein angiography findings in the optic disc: The absence of significant abnormal findings in the optic disc suggests that the occlusion is not due to a central retinal vein occlusion.<br>Based on these findings, the likely cause of the branch retinal vein occlusion in the left eye is a combination of venous congestion, leakage from the microvasculature, and cystoid macular edema.<br>[No information about common causes associated with BRVO, such as thrombosis, arteriosclerosis, hypertension, and diabetes. ] |
| Harmfulness | **Finding:** There was an arteriovenous crossing sign beneath the optic disc, resulting in the distal branch vein showing delayed reflux in its side branch. Within the affected area, there was venous congestion and leakage from the microvasculature, leading to the formation of numerous small non-perfused areas. Additionally, there was cystoid macular edema present along the arcades and its adjacent and below margins in the left eye. No significant abnormal fluorescence was observed in the optic disc of the left eye. **Impression:** Branch retinal vein occlusion Left eye. | What should I do? | Based on the findings of the retinal vein occlusion in your left eye, it is important to seek immediate medical attention from an ophthalmologist. This condition can cause vision loss if left untreated, so it is crucial to receive prompt medical care.<br>Here are some potential treatment options for branch retinal vein occlusion:<br>1. Observation: In some cases, the occlusion may resolve on its own within a few days to weeks without any treatment. However, close monitoring by an ophthalmologist is necessary to ensure that the vision does not worsen.<br>2. Intravitreal injections: Injecting a medication called triamcinolone into the vitreous cavity can help reduce the swelling and inflammation caused by the occlusion. This can help improve vision and prevent further damage.<br>3. Laser treatment: A laser can be used to create a channel in the occluded vein to help restore blood flow. This can help improve vision and prevent further damage.<br>4. Vitrectomy: In severe cases, a vitrectomy may be necessary to remove the blood and debris from the vitreous cavity. This can help improve vision and prevent further damage.<br>It is important to note that these treatments are not always successful and may not restore vision completely. However, they can help slow down or stop the progression of vision loss.<br>In addition to these medical treatments, there are some things you can do at home to help manage the symptoms of branch retinal vein occlusion: |

| | | | |
|---|---|---|---|
| | | | 1. Rest: Resting your eyes and avoiding any strenuous activities can help reduce eye strain and fatigue.<br>2. Cold compress: Applying a cold compress to the affected eye can help reduce swelling and discomfort.<br>3. Eye drops: Using artificial tears or other eye drops can help keep the eye moist and reduce discomfort.<br>4. Light therapy: Exposure to bright light can help reduce the risk of further vision loss.<br>It is important to work closely with your ophthalmologist to determine the best course of treatment for your specific condition. With prompt and appropriate treatment, many people with branch retinal vein occlusion can recover some or all of their vision. |
| Satisfaction | **Finding**: Left eye fundus fluorescein angiography (FFA) findings: Throughout the procedure, there is weak fluorescence observed due to the detachment of the neuroepithelium in the central macula. Above the central macula, a focal area of pigment epithelial leakage is seen, exhibiting hyperfluorescence. No significant abnormal fluorescence is observed in the peripheral retina. There are no apparent abnormal fluorescence findings in the optic disc.<br>**Impression**: Central serous chorioretinopathy in the left eye. | What should I do? | Based on the findings of the left eye fundus fluorescein angiography (FFA), there are a few potential next steps that you could consider:<br>1. Monitor the patient closely: The weak fluorescence observed in the central macula and the focal area of pigment epithelial leakage may be indicative of a chronic central serous chorioretinopathy (CSC) episode. It is important to closely monitor the patient's vision and retinal status over time to see if the condition worsens or improves.<br>2. Consider photodynamic therapy (PDT): PDT may be a useful treatment option for CSC. This involves the administration of a light-sensitive medication, followed by exposure to a low-intensity laser. The laser energy activates the medication, which then helps to reduce the leakage of fluid and pigment in the retina.<br>3. Watchful waiting: If the patient's vision is stable and there are no significant changes in the retina, a wait-and-watch approach may be appropriate. This involves closely monitoring the patient's condition over time but without immediate treatment.<br>4. Consider other treatments: In addition to PDT, other treatments for CSC may include corticosteroids, anti-vascular endothelial growth factor (VEGF) injections, and retinal photocoagulation. The most appropriate treatment will depend on the severity and location of the CSC episode, as well as the patient's overall health and medical history.<br>It is important to consult with a retinal specialist or ophthalmologist to determine the best course of treatment for the patient based on their individual case. |

**Supplementary Table 5.** Comparison of FFA-GPT and ChatFFA[1].

| | **ChatFFA** | **FFA-GPT** |
|---|---|---|
| **Method** | BLIP | BLIP+LlaMA 2 |
| **Top-1 Accuracy** | 0.94 | 0.91 |
| **Top-1 F1 score** | 0.87 | 0.82 |
| **BLEU-4** | 0.26 | 0.34 |
| **CIDEr** | 0.23 | 0.33 |
| **ROUGE** | 0.30 | 0.36 |
| **Data used for model development** | 9,392 reports and 4,110,581 QA pairs | 9,392 reports |
| **Training time** | 9 days | 3 days |

1. Chen X, Xu P, Li Y, et al. ChatFFA: Interactive Visual Question Answering on Fundus Fluorescein Angiography Image Using ChatGPT. 2023-09-22 2023;doi:10.2139/ssrn.4578568

**Supplementary Table 6. The question lists for question answering.**

| | |
|---|---|
| 1. | Is there any abnormality? |
| 2. | What is the diagnosis? |
| 3. | Where is the abnormality? |
| 4. | Is this condition severe? |
| 5. | Can this condition cause blindness? |
| 6. | Will this condition progress? |
| 7. | What is the outcome of this condition? |
| 8. | What is the prognosis for this condition? |
| 9. | What might be the cause of this condition? |
| 10. | What are some common complications that can occur? |
| 11. | What should I do? |
| 12. | What is your suggestion for me? |
| 13. | What kind of further testing might be necessary? |
| 14. | What might be the treatment options for this condition? |
| 15. | How can I prevent this condition? |
| 16. | Can you explain how FFA works and what it can detect? |
| 17. | What is the arterial/venous/late phase of FFA? |
| 18. | How long does an FFA test usually take to complete? |
| 19. | Is there anything I should avoid doing after an FFA test? |
| 20. | Will the contrast dye used in angiography remain in my body? |
